# Interpreting one oral health impact profile point

**DOI:** 10.1186/1477-7525-11-12

**Published:** 2013-01-30

**Authors:** Daniel R Reissmann, Ira Sierwald, Guido Heydecke, Mike T John

**Affiliations:** 1 Department of Prosthetic Dentistry, Center for Dental and Oral Medicine, University Medical Center Hamburg-Eppendorf, Martinistrasse 52, D-20246, Hamburg, Germany; 2 Department of Diagnostic and Biological Sciences, University of Minnesota, 515 Delaware Street SE, Minneapolis 55455, MN, USA; 3 Division of Epidemiology and Community Health, University of Minnesota, 1300 S Second Street, Minneapolis 55454, MN, USA

**Keywords:** OHIP, Response format, OHRQoL, Assessment

## Abstract

**Background:**

Interpretation of scores from oral health-related quality of life (OHRQoL) instruments, such as the Oral Health Impact Profile (OHIP) is challenging. It was the aim of this study to determine how many oral impacts correspond to one point of the 49-item OHIP using a new approach which translates numeric problem counts into the traditionally used ordinal OHIP response categories.

**Methods:**

A sample of 145 consecutively recruited prosthodontic patients seeking treatment or having a routine examination completed the German version of the 49-item OHIP with the original ordinal response format as a self-administered questionnaire. In addition, the numerical frequencies of impairment during the previous month were requested in personal interviews. Based on a multilevel mixed-effects linear regression, we estimated the mean difference with 95% confidence interval (CI) in numerical frequency between two adjacent ordinal responses.

**Results:**

A numerical frequency of 15.2 (CI: 14.8 – 15.7) impacts per month corresponded to one OHIP point. This translates to approximately one impact every other day in the past month.

**Conclusions:**

The oral problem count per day that corresponds to one OHIP-49 point can be used to interpret this instrument’s scores in cross-sectional and longitudinal studies. This number can help to better understand OHRQoL burden for patients, clinicians, and researchers alike.

## Introduction

During the past thirty years, oral health-related quality of life (OHRQoL) instruments summarizing different aspects of perceived oral health in a composite score have become the standard to measure the impact of oral disease and dental interventions [1-7]. As these instruments assess what directly matters to the patient (patient-oriented or patient-reported outcomes [PRO]), they are conceptually superior in assessing disease and intervention impact on patients’ perceived oral health compared to disease-oriented measures such as pocket depth or plaque accumulation, which express their importance indirectly through PRO.

Unfortunately, the conceptual advantage of PROs is accompanied by a technical challenge: OHRQoL scores are difficult to interpret because they come in a metric that is unfamiliar. To become informative they need a framework for interpretation. Consequently, reference values, *i.e*., typical scores for target populations of the instrument, are used. Reference values provide context information, usually for single scores, because they compare an individual’s or a group’s standing to a population of interest. Change scores (differences of scores) often assess treatment effects. Here, the minimal important difference is frequently used to give interpretation what magnitude of change is relevant for individuals [8,9].

The Oral Health Impact Profile is the most widely used and methodologically most investigated OHRQoL instrument. Hence, both norms and minimal important differences are available [10-18]. However, the relationship of OHIP scores to countable oral health impacts [19] provides another opportunity to interpret both single *and* change scores. To know how many oral impacts correspond to one OHIP point is informative for researchers, clinicians, and patients alike. This value can be used as base frequency to be multiplied by the actual OHIP score. The resulting problem count translates OHRQoL burden into an easy to interpret number that is informative about the magnitude of impacts perceived by the individual.

It was the aim of this study to determine how many oral impacts correspond to one point of the 49-item Oral Health Impact Profile.

## Methods

### Study design, setting and subjects

A sample of 145 consecutive adult prosthodontic patients (21 to 88 yr; mean age ± SD: 58.5 ± 14.9 yr; 54% females) was recruited for this cross-sectional study at the Department of Prosthetic Dentistry of the University Medical Center Hamburg-Eppendorf, Hamburg, Germany. We did not have a pilot of numerical response data, thus, there was no opportunity to perform a formal sample size calculation.

All adult patients who had an appointment for a routine examination or for treatment in February 2010 were included. Patients who could not follow the questionnaire because of comprehension or language difficulties were excluded. For the analysis, patients were divided into subgroups according to denture status (one patient excluded due to missing value for denture status) - none or only fixed partial dentures (none/FPD; n = 68; 47%) and removable partial dentures or complete dentures (RPD/CD; n = 76; 53%) as well as according to median age (62 yr).

The study protocol was reviewed and approved by the appropriate local Institutional Review Board (PV3280), Ethics Committee of the Medical Council of Hamburg (Ethik-Kommission der Ärztekammer Hamburg). All study participants gave written informed consent.

### Assessment of OHRQoL

For measurement of oral health-related quality of life, the German version (OHIP-G) [20] of the OHIP [13] was applied. The OHIP-G has 49 questions (items) derived from the English-language version and additional four items specific for the German population (‘avoid eating with others’, ‘take longer to complete meal’, ‘joint noises’, ‘dry mouth’) [12]. Each OHIP item elicits information about how frequently subjects experienced a specific impact in the last month. For international comparability, we applied only the 49 items derived from the English-language version in this study.

Subjects completed the OHIP-G with its standard ordinal format (‘never’, ‘hardly ever’, ‘occasionally’, ‘often’, ‘very often’) as a self-administered questionnaire. This was followed by a personal interview performed by a single examiner (IS) in which patients were asked to count how often they had actually experienced the impact of problems referred to in each OHIP item within the last four weeks separately. The examiner (IS) was beforehand trained for the interviews by the first author (DRR) of this manuscript who has conducted several OHIP studies. Interviews were conducted immediately after patients had completed the questionnaire using the same item order and questioning as in the self-administered questionnaire. The impact of items that were previously rated as ‘never’ was set to 0, and these items were not included in the personal interview. Possible responses ranged from ‘once’ to the pre-defined maximum of ‘four times a day’ in the preceding four weeks (as the equivalent to the one month period of the OHIP response format) for each item. Any frequency in between the extreme values was permitted. The responses were transformed to a numerical impact frequency. Thus possible responses such as ‘once a week’ corresponded to 4, ‘three times a week’ to 12, ‘once a day’ to 28, and the maximum of ‘four times a day’ to 112 impacts in the last four weeks.

### Data analysis

The analyses involved the comparison of the two OHIP item scores obtained from the different response formats. The first part of the analysis was performed on the patient level, *i.e.*, we computed summary scores of the ordinal responses and the numerical frequencies for each patient and assessed whether the ordinal OHIP summary scores differed significantly with respect to gender, age group and denture status using Student’s two-sample *t*-test. Additionally, we counted the items rated at least ‘hardly ever’ (any impact) to obtain the number of problems (affected OHIP items) for each patient.

How the numerical impact frequencies were related to the ordinal OHIP responses was analyzed using regression analysis. Because both ordinal and numerical responses were provided by each patient for all 49 OHIP items, we used a multilevel mixed-effects linear regression model where the two random factors ‘patients’ and ‘items’ were crossed. To estimate a mean and 95% confidence interval (CI) for the number of impacts in each ordinal OHIP category, we fitted four separate models for the OHIP response categories ‘hardly ever’ to ‘very often’ as independent variable and the numerical response as the dependent variable. To estimate a difference and CI between neighboring OHIP response categories, we fitted a model using OHIP’s ordinal 0 to 4 response categories as a linear variable and the numerical response as the dependent variable. All 49 OHIP items were included in all regression analyses. The difference of numerical impact frequencies between two adjacent ordinal response categories, indicated by the coefficient in the regression analyses, provided the value of the number of impacts corresponding to one ordinal OHIP point.

Before performing the multi-level model, we assessed whether the relationship between the ordinal responses scale and the numerical frequency was approximately linear. The ordinal values and the corresponding numerical frequencies (patients’ means) were plotted with a locally weighted regression line (Lowess [21]) fitted to the data. Two researchers (MTJ and DRR) independently judged the curve visually to ascertain whether or not a linear relationship between the scores could be assumed. A Spearman rank correlation coefficient was computed between the OHIP standard ordinal response format and the patients’ means of the numerical frequency to provide an estimate of the strength of relationship.

All analyses were performed using the statistical software package stata, Release 12 (Stata Statistical Software, College Station, TX, USA) with the probability of a type I error set at 0.05.

## Results

### Characteristics of study population

OHRQoL was substantially impaired in the study population. Summary scores of ordinal responses ranged from 2 to 129 OHIP points (mean: 41.6; SD: 30.0) and scores of numerical response ranged from 2 to 3167 impacts (mean: 531.7; SD: 600.6). Patients indicated having at least 2 different problems (OHIP items) with a maximum of 48 of 49 possible problems. On average, patients indicated 19.7 (SD: 11.5) different problems.

Female patients had a higher mean ordinal OHIP score than male patients (female: 45.1, SD: 30.4; male: 37.5, SD: 29.2; *t*–test: p = 0.131) and younger patients had slightly higher mean ordinal OHIP scores than older patients (< 62 yr: 44.0, SD: 32.6; > = 62 yr: 39.1, SD: 27.1; *t*–test: p = 0.322). However, both differences in ordinal OHIP scores were not statistically significant. OHRQoL of patients with RPD or with CD was significantly more impaired indicated by higher OHIP scores than patients with no dentures or only FPD (RPD/CD: 47.6, SD: 31.1; non/FPD: 35.4, 27.5; *t*–test: p = 0.014).

### Relationship between ordinal OHIP response and numerical frequencies of impacts

An OHIP response of ‘hardly ever’ corresponded to 9.1 impacts per month, ‘occasionally’ to 19.4 impacts, ‘often’ to 43.0 impacts, and ‘very often’ to 70.1 impacts (Table 1).

**Table 1:** Means and CIs of numerical frequencies corresponding to the ordinal OHIP response categories (#1 - #4) and one OHIP point (#5); results of multi-level regression models with crossed random factors “patients” and “items” (random-effects parameters not presented)

**Model**	**Variable**	**Coefficient**	**95% CI**	**P value**
*# 1*				
	'Hardly ever'	9.1	(7.1 – 11.0)	< 0.001
*# 2*				
	'Occasionally'	19.4	(16.1 – 22.7)	< 0.001
*# 3*				
	'Often'	43.0	(37.0 – 49.0)	< 0.001
*# 4*				
	'Very often'	70.1	(61.6 – 78.6)	< 0.001
*# 5*				
	One OHIP point	15.2	(14.8 – 15.7)	< 0.001

Locally weighted regression analysis ( 1) revealed an approximately linear functional relationship between standard ordinal response scores and those generated by the patients’ means of the numerical response format, supporting the use of statistical analyses depending on linear relationships. Both response formats correlated highly with each another (r = 0.87).

**Figure 1:**
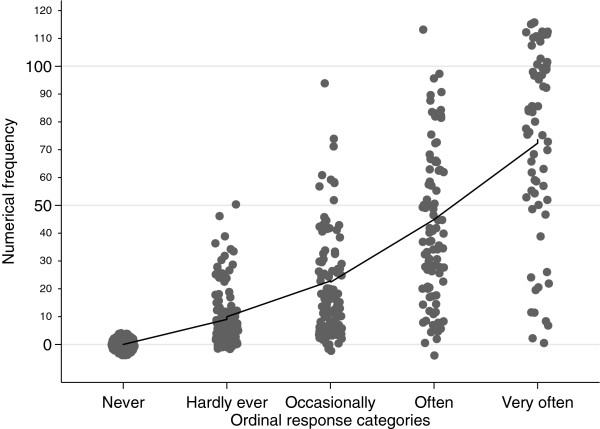
Scatterplot (with spherical random noise) of Oral Health Impact Profile (OHIP) item values of the ordinal response format and the numerical frequencies (patients’ means), with locally weighted regression line (Lowess) fitted to the data.

According to the multilevel model, the mean difference in numerical frequencies between two adjacent ordinal responses was 15.2 (Table 1), *i.e.*, one OHIP point corresponded to approximately 15 problems occurrences.

## Discussion

We found that in prosthodontic patients one OHIP-49 point corresponded to 15.2 impacts over the period of one month. This translates to approximately one impact every other day.

This number may be used to better interpret previously observed OHIP results. For example, prosthodontic patients receiving autologous bone grafts prior to insertion of dental implants would experience about 38 impacts a day when seeking treatment [22]. The clinically relevant difference between patients with FPDs and patients with RPDs would correspond to about 6 impacts a day and the minimal important difference for the OHIP translates to 3 impacts per day [11]. An increase of 1 missing occlusal unit in patients with shortened dental arches would be associated with an increase of 1 impact a day [23]. If this number was generalized to another patient population such as TMD patients (a population with known psychosocial impact) they are likely to experience between 15 and 31 daily impacts, depending on the diagnoses. This is at least three times more often than the mean of 5 daily impacts among general population subjects without TMD [24]. Reference values could be compared across countries, *e.g.*, general population subjects wearing complete dentures in Germany reported daily impacts 4 times as often compared to those subjects in Hungary (median: 12 vs. 3) [12,25]. These numbers give an impression of how frequently OHRQoL problems occur, how patients suffer from oral disease and how they benefit from interventions. Patients, clinicians and researchers have an easy and practical guide to interpret OHRQoL in more detail.

The study population comprised of prosthodontic patients who either had an appointment for an annual examination or for regular treatment. We considered our study population to be typical as they suffered from common dental diseases - caries and periodontitis. The oral status of the patients ranged from only natural teeth, fixed partial dentures or removable partial dentures to complete dentures. Differences in OHRQoL impairment with respect to gender and age group were not statistically significant and, therefore, might be due to chance. However, despite the fact that our patients experienced a wide spectrum of oral health problems, it is not clear how far our results can be generalized to other populations. It might be that the oral impacts corresponding to one point of the OHIP are different in populations with lower impaired OHRQoL. In our study, we included both patients with highly impaired OHRQoL indicated by treatment needs and patients with less impaired OHRQoL at their annual dental check-up.

We did not encounter any difficulties when applying the OHIP in the personal interview. However, since patients had to think about the exact frequency of each item (and not only making a estimation on a ordinal 5-point scale), the interviews with the numerical impact frequencies as the response scale took longer compared to the self-administered completion of the questionnaire. Counting oral health impacts seems easy, but in practice it is actually quite challenging [19]. This is a reason why the counting method using the OHIP numerical response format is not very practical for most settings. Based on our clinical experience, we believe that it may be interesting for settings where the specific number of oral health problems is small but the impacts are severe.

Our methodological approach has strengths and limitations. To calculate the mean number of impacts for each OHIP response category is sound and does not rest on many assumptions. We considered the OHIP items to be interchangeable indicators of one construct and there is some evidence for this assumption from factor analytic studies showing that OHIP has a dominating general factor even if the construct is considered multidimensional [26]. In contrast, fitting a straight line through OHIP’s five ordinal response categories and deriving one problem count per OHIP point rests on several assumptions. In addition to the one mentioned above, it is assumed that the relationship between ordinal and numerical OHIP values is linear, that the difference between the ordinal categories is equal and that the straight line fits reasonably well through the individual data points to name major points. Although we found some evidence that supported these assumptions, our results should nevertheless be interpreted with caution. The distributions of the numerical frequencies corresponding to the ordinal responses were of a substantial magnitude. However, this has already been observed previously in other studies investigating the relationship between ordinal and numerical responses, *e.g.*, pain ratings on an ordinal scale and a visual analogue scale [27]. We condensed a complex phenomenon into one number and our results are intended as first step into an innovative interpretation of OHIP scores. Nevertheless, we believe using this single number is worthwhile as it is a simple and practical guide to interpret OHIP scores. As OHIP scores are potentially influenced by recall periods [20,28], memory effects [18], order effects [15,16], and administration method [17]; these methodological factors may also potentially influence the problem count per OHIP point. However, it has been shown that OHIP scores are rather robust against the influence of methodological factors. Therefore, there is no compelling evidence why the problem count per OHIP point should be substantially influence by methodological factors.

A more challenging question is whether our results can be informative for the several available OHIP short forms. We assume that the number of oral impacts for one OHIP point of a single item (problem) should be identical in long and short forms. For summary scores, extrapolation of our results rests on the assumption that OHIP has a dominating underlying general factor and that all items are basically interchangeable. Under this assumption and using OHIP-5 as an example, this would result in a summary score of approximately one-tenth of the OHIP-49 if administered simultaneously, and the number of oral impacts for one point of the OHIP-5 summary score should be tenfold the number for the OHIP-49 to yield comparable results. For the OHIP-14 summary score, the number of oral impacts for one OHIP point should be multiplied with 3.5, respectively. Such results should be carefully checked whether they fit with expectations and other findings.

## Conclusion

Our approach to link OHIP scores to actual numbers of OHIP counts is new and summarizing our results in one number for impacts per day is a simplification of a complex phenomenon. The intent is to provide a pragmatic help for OHIP score interpretation – a key attribute for quality of life measures [29] and one of the most important steps to make OHRQoL findings clinically relevant.

## Abbreviations

OHIP: Oral Health Impact Profile; OHRQoL: Oral Health-Related Quality of Life; FPD: Fixed Partial Denture; RPD: Removable Partial Denture; CD: Complete Denture; CI: 95% Confidence Interval; PRO: Patient-Reported Outcome.

## Competing interests

The authors declare that they have no competing interests.

## Authors’ contribution

All authors participated in the design and coordination of the study. IS collected the data. DRR and MTJ performed the statistical analyses. DRR drafted the manuscript with the help of IS, MTJ and GH. All authors read and approved the final manuscript.
